# Natural Language Processing of Clinical Documentation to Assess Functional Status in Patients With Heart Failure

**DOI:** 10.1001/jamanetworkopen.2024.43925

**Published:** 2024-11-07

**Authors:** Philip Adejumo, Phyllis M. Thangaraj, Lovedeep Singh Dhingra, Arya Aminorroaya, Xinyu Zhou, Cynthia Brandt, Hua Xu, Harlan M. Krumholz, Rohan Khera

**Affiliations:** 1 Section of Cardiovascular Medicine, Department of Internal Medicine, Yale School of Medicine, New Haven, Connecticut; 2 VA Connecticut Healthcare System, West Haven; 3 Section of Health Informatics, Department of Biostatistics, Yale School of Public Health, New Haven, Connecticut; 4 Department of Health Policy and Management, Yale School of Public Health, New Haven, Connecticut; 5 Center for Outcomes Research and Evaluation, Yale-New Haven Hospital, New Haven, Connecticut; 6 Section of Biomedical Informatics and Data Science, Yale School of Medicine, New Haven, Connecticut

## Abstract

**Importance:**

Serial functional status assessments are critical to heart failure (HF) management but are often described narratively in documentation, limiting their use in quality improvement or patient selection for clinical trials.

**Objective:**

To develop and validate a deep learning natural language processing (NLP) strategy for extracting functional status assessments from unstructured clinical documentation.

**Design, Setting, and Participants:**

This diagnostic study used electronic health record data collected from January 1, 2013, through June 30, 2022, from patients diagnosed with HF seeking outpatient care within 3 large practice networks in Connecticut (Yale New Haven Hospital [YNHH], Northeast Medical Group [NMG], and Greenwich Hospital [GH]). Expert-annotated notes were used for NLP model development and validation. Data were analyzed from February to April 2024.

**Exposures:**

Development and validation of NLP models to detect explicit New York Heart Association (NYHA) classification, HF symptoms during activity or rest, and frequency of functional status assessments.

**Main Outcomes and Measures:**

Outcomes of interest were model performance metrics, including area under the receiver operating characteristic curve (AUROC), and frequency of NYHA class documentation and HF symptom descriptions in unannotated notes.

**Results:**

This study included 34 070 patients with HF (mean [SD] age 76.1 [12.6] years; 17 728 [52.0]% female). Among 3000 expert-annotated notes (2000 from YNHH and 500 each from NMG and GH), 374 notes (12.4%) mentioned NYHA class and 1190 notes (39.7%) described HF symptoms. The NYHA class detection model achieved a class-weighted AUROC of 0.99 (95% CI, 0.98-1.00) at YNHH, the development site. At the 2 validation sites, NMG and GH, the model achieved class-weighted AUROCs of 0.98 (95% CI, 0.96-1.00) and 0.98 (95% CI, 0.92-1.00), respectively. The model for detecting activity- or rest-related symptoms achieved an AUROC of 0.94 (95% CI, 0.89-0.98) at YNHH, 0.94 (95% CI, 0.91-0.97) at NMG, and 0.95 (95% CI, 0.92-0.99) at GH. Deploying the NYHA model among 182 308 unannotated notes from the 3 sites identified 23 830 (13.1%) notes with NYHA mentions, specifically 10 913 notes (6.0%) with class I, 12 034 notes (6.6%) with classes II or III, and 883 notes (0.5%) with class IV. An additional 19 730 encounters (10.8%) could be classified into functional status groups based on activity- or rest-related symptoms, resulting in a total of 43 560 medical notes (23.9%) categorized by NYHA, an 83% increase compared with explicit mentions alone.

**Conclusions and Relevance:**

In this diagnostic study of 34 070 patients with HF, the NLP approach accurately extracted a patient’s NYHA symptom class and activity- or rest-related HF symptoms from clinical notes, enhancing the ability to track optimal care delivery and identify patients eligible for clinical trial participation from unstructured documentation.

## Key Points

**Question:**

Can a deep learning natural language processing (NLP) approach accurately extract New York Heart Association (NYHA) functional classification and activity- or rest-related heart failure (HF) symptoms from unstructured clinical notes, a class I recommendation in HF guidelines?

**Findings:**

In this diagnostic study including 34 070 patients with HF, deep learning NLP models accurately extracted NYHA class and activity- or rest-related symptoms from clinical notes, with class-weighted area under receiver operator characteristic curves of 0.98 to 0.99 and 0.94 to 0.95, respectively. Deploying the models on 182 308 outpatient medical notes identified 13.1% notes with explicit NYHA mentions and an additional 10.8% encounters with activity- or rest-related symptoms categorized into NYHA classes.

**Meaning:**

These findings suggest that a computable NLP-based approach to functional status assessments may enhance the ability to track guideline-directed medical therapy and identify clinical trial–eligible patients from unstructured documentation.

## Teaser

This diagnostic study describes the development and validation of a deep learning natural language processing strategy for extracting functional status assessments from unstructured clinical documentation among patients with heart failure (HF).

## Introduction

Heart failure (HF) is characterized by broad symptoms that impair patients’ functional ability and adversely affect their quality of life.^1,2^ Clinical practice guidelines recommend regular functional status evaluations using the New York Heart Association (NYHA) classification system, which grades HF severity based on limitations in physical activity and associated symptoms.^3,4^ These assessments are crucial for informing therapeutic decisions, such as selecting appropriate guideline-directed medical therapy and determining the need for primary prevention implantable cardioverter defibrillators (ICDs).^5^

While regular functional status assessment measurements are expected to be a standard component of clinical management for patients with HF, these assessments are primarily documented in unstructured medical notes.^6,7,8^ The impact of these assessments on treatment decisions remains understudied due to persistent challenges of manually reviewing clinical records.^9,10^ This further impedes downstream applications of clinical assessment, such as the automated evaluation guideline-directed care quality and the scalable recruitment from the electronic health record (EHR) for clinical trials studying HF.

To address this, we developed and validated efficient, transformer-based deep learning models that use natural language processing (NLP) to automate the extraction of recorded functional status assessments within unstructured clinical notes.^11^ This study developed a scalable, artificial intelligence–driven approach that can reliably identify explicit mentions of NYHA classes and categorize relevant functional status descriptors within the vast array of unstructured text in the EHR.^12^

## Methods

The Yale University institutional review board reviewed the study, approved the protocol, and waived the need for informed consent because this is a secondary analysis of existing data. This study adheres to both the Strengthening the Reporting of Observational Studies in Epidemiology (STROBE) reporting guideline and the Standards for Reporting of Diagnostic Accuracy (STARD) reporting guideline.

### Data Source

We used Yale New Haven Health System (YNHHS) EHR data from 2013 to 2022, encompassing an academic hospital and a community practice network with geographically distinct sites and separate clinician panels. YNHHS caters to a diverse demographic and serves a community that reflects the national US population across age, sex, race, ethnicity, and socioeconomic status.^13^ We extracted key structured and unstructured EHR data for the study population from the YNHHS Epic Clarity database. The structured fields included patient demographics, diagnosis, procedure codes, ejection fraction (EF), and health care encounters. The unstructured data included medical notes, which provide a comprehensive clinical history for each patient.

### Study Population

The study cohort comprised patients diagnosed with HF who had at least 1 health care encounter within any of the YNHHS Heart and Vascular Center Outpatient Practices affiliated with the academic medical center, Yale New Haven Hospital (YNHH), the community-based practice, Northeast Medical Group (NMG), and the community-based teaching hospital, Greenwich Hospital (GH), between January 1, 2013, and June 30, 2022 (eFigure 1 and eTable 1 in Supplement 1). Eligible patients were 18 years or older and had 1 or more health care encounters with an *International Classification of Diseases, Ninth Revision, Clinical Modification (ICD-9-CM)* and *International Statistical Classification of Diseases, Tenth Revision, Clinical Modification (ICD-10-CM)* code for HF. We also identified comorbidities, including acute myocardial infarction, cardiomyopathy, hypertension, diabetes, and chronic kidney disease, using the relevant diagnosis codes (eFigure 2 and eTable 2 in Supplement 1). Medical documentation, including history and physicals, progress notes, referral letters, and assessments and plans, were examined at the encounter level. This method comprehensively captured elements reflecting the patient’s health and functional status and treatment adjustments recorded by different health care providers across multiple visits.

### Study Outcomes

We assessed 3 outcomes of interest. We evaluated our deep learning models’ performance in identifying explicit mentions of NYHA classes in unstructured medical notes against expert EHR abstraction, the performance of a model designed to extract activity or rest-related HF symptoms based on expert annotation of the notes, and a descriptive evaluation of functional status assessment frequency across all outpatient encounters.

### Manual Annotation

Annotators labeled 2000 randomly selected outpatient notes at YNHH for the specified document classification task. In addition, we annotated a separate set of 1000 notes from outpatient clinics at GH and NMG for additional external validation (500 each) (eFigure 3 and eMethods in Supplement 1). Each note was labeled for explicit mentions of NYHA symptom class and the presence of HF symptoms associated with activity or rest (eTable 3 in Supplement 1). Three expert annotators (P.A., L.S.D., and A.A.) collaboratively established the criteria for class identification and symptom extraction. Annotations were completed at the sentence level and aggregated at the note level to ensure a standardized and precise approach across the dataset. Additional details on the data preprocessing can be found in the eMethods in Supplement 1).

### Model Development

We randomly separated the annotated dataset from YNHH (the development site) into 3 subsets: 80% was allocated for training the models, 10% for validation to fine-tune the hyperparameters, and the remaining 10% for internal testing. This split is a standard practice in machine learning, providing sufficient data for training, model validation, and unbiased performance assessment, respectively.^14^ We used a transfer learning approach to fine-tune publicly available ClinicalBERT-based model weights, which had been specifically pretrained on a large corpus of clinical text (eMethods in Supplement 1).^15^ The fine-tuning process involves adjusting ClinicalBERT model weight parameters to better capture the terminology specific to our 2 independent document classification tasks: distinguishing among the various NYHA functional status classes (class I, class II, class III, class IV, or no mention of NYHA class) and identifying the documented symptoms of HF occurring during activity (symptoms with activity, lack of symptoms with activity, or no mention) or at rest (symptoms with rest, lack of symptoms with rest, or no mention) (eMethods in Supplement 1).

### Interpretability Analysis

We conducted an interpretability analysis using an adapted version of the Shapley Additive Explanations (SHAP) method (eMethods in Supplement 1).^16,17^ We selected a stratified sample of 100 notes from each NYHA class to reflect a broad spectrum of clinical scenarios. Using the SHAP Explainer, we permuted features within these notes to create 2000 synthetic examples, and the resulting SHAP values for these permutations provided insight into the significance of individual words and phrases in the notes. We calculated and aggregated these SHAP values to assess the mean positive impact of each feature, enabling us to determine the most influential factors in our model’s decision-making process.

### Model-Defined Prevalence of Functional Status Assessments

To demonstrate the clinical utility of our models, we evaluated the frequency of NYHA class documentation and descriptions of activity- or rest-related HF symptoms in the broader set of unannotated EHR notes from YNHHS that were not included in model development or validation. We deployed the trained model to identify explicit mentions of NYHA classes and the presence of activity- or rest-related symptoms in these records. Furthermore, using identified activity- or rest-related symptoms, we recategorized patients without explicit mentions of NYHA class in their clinical documentation into corresponding NYHA classes. For this, symptoms at rest were considered indicative of NYHA class IV, while symptoms with varying levels of activity were mapped to classes I to III based on the severity and context of the reported symptoms (eFigure 4 in Supplement 1). By combining the explicit mentions identified by the NYHA class detection model and the additional classifications provided by the model for detecting activity- or rest-related symptoms, we sought to enhance the capture of functional status information and provide a more comprehensive assessment of HF severity across the patient population.

To further illustrate the potential impact of our models, we assessed the frequency of NYHA class documentation in the year preceding the implantation of an ICD, a key HF therapy for which NYHA class assessment is required in determining the eligibility in clinical practice guidelines.^18^ We identified patients at YNHH who underwent ICD implantation using the relevant procedure codes and determined the presence of explicit NYHA class mentions in their clinical notes the year before the procedure (eTable 4 in Supplement 1). This analysis aimed to showcase the utility of our models in identifying clinically relevant information during critical windows of care.

### Statistical Analysis

For all descriptive analyses, categorical variables were reported as frequency and percentage, while continuous variables were summarized as mean and SD. Given the multiclass nature of our analysis, we assessed their performance using both micro-averaged and macro-averaged metrics (eMethods in Supplement 1). Study metrics include the area under the receiver operating characteristic curve (AUROC), which assesses overall discriminative ability; area under the precision-recall curves (AUPRC), a suitable metric for imbalanced datasets that combines positive predictive value (precision) and sensitivity (recall); accuracy, a measure of overall correctness; precision and recall, which indicate reliability of positive predictions and ability to identify all positive cases, respectively; specificity, indicating ability to correctly identify negative cases; and F_1_-score, the harmonic mean of precision and recall.^19^ The models produced continuous probabilities, which we dichotomized for the presence or absence of the condition by thresholds that maximized Youden Index, ensuring a balance between sensitivity and specificity. For each of these metrics, 95% CIs were obtained from bootstrap resampling with 1000 iterations. To assess the model’s performance across diverse populations, we conducted subgroup analyses based on race and ethnicity, sex, and EF. Race and ethnicity were self-reported by patients during medical encounters, with race categorized as American Indian or Alaska Native, Asian, Black, Native Hawaiian or Other Pacific Islander, White, unknown, or other (ie, patients who identified with more than 1 race, races not listed separately, or patients whose race was not recorded), and ethnicity categorized as Hispanic or Latino, non-Hispanic, or unknown. We evaluated the model’s accuracy, sensitivity, specificity, and AUROC for each subgroup. Patients were categorized into 2 groups based on their most recent EF measurement: reduced (EF <40%) and preserved or mildly reduced EF (EF ≥40%).

Analyses were conducted using Python version 3.10 (Python Software Foundation). Data were analyzed from February to April 2024. 

## Results

### Study Cohort

There were 34 070 patients with 1 or more health care encounters at cardiovascular outpatient practices within YNHHS between January 1, 2013, and June 30, 2022, and a recorded diagnosis of HF. This included 29 555 patients at YNHH (168 842 encounters; 168 655 clinical notes), 2526 patients at NMG (4415 encounters; 4435 notes), and 1989 patients at GH (7733 encounters; 12 218 notes). The mean (SD) age of the cohort was 76.1 (12.6) years, with 17 728 (52.0%) female. A total of 105 patients (0.3%) were American Indian or Alaska Native, 434 patients (1.3%) were Asian, 4422 patients (12.9%) were Black, 57 patients (0.2%) were Native Hawaiian and Other Pacific Islander, and 27 109 (79.5%) patients were White; 2060 patients (6.0%) were Hispanic or Latino and 31509 (92.5%) patients were not Hispanic or Latino. Among these, 28 994 patients (85.1%) had a diagnosis of hypertension, 14 580 patients (42.8%) had diabetes, and 10 648 patients (31.3%) had chronic kidney disease (Table 1).

**Table 1.** Demographic Characteristics Across Yale-New Haven Hospital, Northeast Medical Group, and Greenwich Hospital

Characteristic	Patients, No. (%)		
	Yale-New Haven Hospital	Northeast Medical Group	Greenwich Hospital
Age, mean (SD), y	76.57 (13.45)	73.64 (12.85)	79.07 (11.54)
Sex			
Male	14 171 (47.9)	1093 (43.3)	1078 (54.2)
Female	15 384 (52.1)	1433 (56.7)	911 (45.8)
Race			
American Indian or Alaska Native	93 (0.3)	12 (0.5)	0
Asian	345 (1.2)	35 (1.4)	54 (2.7)
Black	4245 (14.4)	106 (4.2)	71 (3.6)
Native Hawaiian or Other Pacific Islander	48 (0.2)	2 (0.1)	7 (0.4)
White	23 147 (78.3)	2216 (87.7)	1746 (87.8)
Unknown	435 (1.5)	79 (3.1)	23 (1.2)
Other^a^	1242 (4.2)	76 (3.0)	88 (4.4)
Ethnicity			
Hispanic or Latino	1873 (6.3)	92 (3.6)	95 (4.8)
Not Hispanic or Latino	27 295 (92.4)	2338 (92.6)	1876 (94.3)
Unknown	387 (1.3)	96 (3.8)	18 (0.9)
Demographics, No.			
Patients	29 555	2526	1989
Encounters	168 424	4415	7733
Medical notes	168 655	4435	12 218
Comorbid conditions			
Acute myocardial infarction	4435 (15.0)	288 (11.4)	222 (11.2)
Cardiomyopathy	9451 (32.0)	955 (37.8)	592 (29.8)
Hypertension	25 106 (84.9)	2159 (85.5)	1729 (86.9)
Diabetes	12 951 (43.8)	926 (36.7)	703 (35.3)
Chronic kidney disease	9530 (32.2)	662 (26.2)	456 (22.9)
Ejection fraction			
No documented ejection fraction	1755 (5.9)	146 (5.8)	113 (5.7)
<40%	6298 (21.3)	552 (21.9)	437 (22.0)
≥40%	21 502 (72.8)	1828 (72.4)	1439 (72.4)

^a^ Includes patients who identified with more than 1 race, races not listed separately, or patients whose race was not recorded.

### Manual Annotation

Of 2000 clinical notes from YNHH that were annotated by experts, 271 (13.6%) had any mention of NYHA class, with 57 (2.9%) class I, 118 (5.9%) class II, 86 (4.3%) class III, and 10 (0.5%) class IV, while 1729 notes (86.4%) did not mention NYHA class (eFigure 5 and eTable 5 in Supplement 1). Descriptions of HF symptoms were reported in 913 notes (45.7%), with activity-related symptoms of HF reported in 486 notes (24.3%), and 329 notes (16.5%) reporting the absence of symptoms with activity. HF symptoms at rest were noted in 45 notes (2.3%), and the lack of symptoms at rest was reported in 53 notes (2.7%) (eFigure 6 and eTable 6 in Supplement 1). Of 500 expert annotated notes from NMG and GH, NYHA classes were explicitly mentioned in 80 notes (16.0%) and 23 notes (4.6%) notes, respectively (eFigure 5 and eTable 5 in Supplement 1). In addition, descriptions of HF-related symptoms during activity were reported in 125 notes (25.0%) and 54 notes (10.8%) notes at NMG and GH, respectively. The absence of symptoms with activity was found in 54 notes (10.8%) and 38 notes (7.6%) of the notes at NMH and GH, respectively (eFigure 6 and eTable 6 in Supplement 1).

### Model Evaluation

The NYHA classification model demonstrated robust performance in identifying the presence of NYHA class in documentation, with a micro-averaged AUROC of 0.99 (95% CI, 0.98-1.00) at YNHH, 0.98 (95% CI, 0.96-1.00) at NMG, and 0.98 (95% CI, 0.92-1.00) at GH (Table 2; eFigure 7 in Supplement 1). The corresponding AUPRCs ranged from 0.85 to 0.96 (Table 2). Similarly, the model performance for classifying individual NYHA classes ranged between 0.96 and 1.00 for classes I, II, III, and IV at YNHH (eTable 7 in Supplement 1). The activity- and rest-related symptom model demonstrated high discriminative ability across all sites, with micro-averaged AUROCs of 0.94 (95% CI, 0.89-0.98) at YNHH, 0.94 (95% CI, 0.91-0.97) at NMG, and 0.95 (95% CI, 0.92-0.99) at GH. The corresponding AUPRCs ranged from 0.83 to 0.88 (Table 2; eFigure 7 in Supplement 1). For activity- and rest-related symptoms, the AUROCs were 0.98 (95% CI, 0.96-0.99) and 0.94 (95% CI, 0.92-0.96), respectively (eTable 7 in Supplement 1).

**Table 2.** Performance Metrics of NLP Models in Classifying Each Functional Status Category

Validation Site	Accuracy (95% CI)	Precision (95% CI)	Recall (95% CI)	Specificity (95% CI)	AUROC (95% CI)	AUPRC (95% CI)	F_1_-Score (95% CI)
**NYHA class model**							
Yale New Haven Hospital							
Micro-average	0.98 (0.96-1.00)	0.98 (0.96-1.00)	0.98 (0.96-1.00)	0.96 (0.96-1.00)	0.99 (0.98-1.00)	0.88 (0.84-0.93)	0.69 (0.63-0.76)
Macro-average	0.98 (0.96-1.00)	0.58 (0.53-0.63)	1.00 (1.00-1.00)	0.98 (0.96-1.00)	0.99 (0.93-1.00)	0.58 (0.53-0.63)	0.69 (0.65-0.74)
Northeast Medical Group							
Micro-average	0.97 (0.96-0.99)	0.97 (0.96-0.99)	0.97 (0.96-0.99)	0.98 (0.96-0.99)	0.98 (0.96-1.00)	0.96 (0.95-0.98)	0.70 (0.66-0.74)
Macro-average	0.97 (0.96-0.99)	0.64 (0.61-0.67)	0.98 (0.97-0.99)	0.98 (0.96-0.99)	0.98 (0.93-1.00)	0.63 (0.60-0.66)	0.74 (0.71-0.77)
Greenwich Hospital							
Micro-average	0.99 (0.98-1.00)	0.99 (0.98-1.00)	0.99 (0.98-1.00)	0.99 (0.99-1.00)	0.98 (0.92-1.00)	0.85 (0.81-0.88)	0.54 (0.49-0.58)
Macro-average	0.99 (0.98-1.00)	0.56 (0.54-0.58)	0.89 (0.88-0.90)	0.99 (0.99-1.00)	0.94 (0.79-1.00)	0.45 (0.41-0.49)	0.58 (0.54-0.61)
**Activity- and rest-related symptom model**							
Yale New Haven Hospital							
Micro-average	0.95 (0.91-0.99)	0.89 (0.83-0.95)	0.91 (0.85-0.97)	0.96 (0.93-1.00)	0.94 (0.89-0.98)	0.83 (0.76-0.90)	0.90 (0.84-0.96)
Macro-average	0.95 (0.91-0.99)	0.87 (0.81-0.94)	0.71 (0.62-0.80)	0.95 (0.90-0.99)	0.83 (0.75-0.90)	0.65 (0.55-0.74)	0.75 (0.67-0.84)
Northeast Medical Group							
Micro-average	0.95 (0.92-0.97)	0.89 (0.85-0.92)	0.91 (0.88-0.95)	0.96 (0.94-0.98)	0.94 (0.91-0.97)	0.83 (0.79-0.88)	0.90 (0.86-0.94)
Macro-average	0.95 (0.92-0.97)	0.84 (0.80-0.88)	0.75 (0.70-0.80)	0.94 (0.91-0.97)	0.85 (0.80-0.89)	0.66 (0.60-0.72)	0.78 (0.73-0.83)
Greenwich Hospital							
Micro-average	0.97 (0.94-0.99)	0.93 (0.90-0.97)	0.93 (0.89-0.97)	0.98 (0.96-1.00)	0.95 (0.92-0.99)	0.88 (0.83-0.94)	0.93 (0.89-0.97)
Macro-average	0.97 (0.94-0.99)	0.84 (0.78-0.90)	0.76 (0.70-0.83)	0.97 (0.95-1.00)	0.87 (0.82-0.92)	0.69 (0.62-0.77)	0.79 (0.73-0.85)

Abbreviations: AUPRC, area under the precision-recall curves; AUROC, area under the receiver operating characteristic curve; NLP, natural language processing; NYHA, New York Heart Association.

### Interpretability Analysis

**Figure 1.**
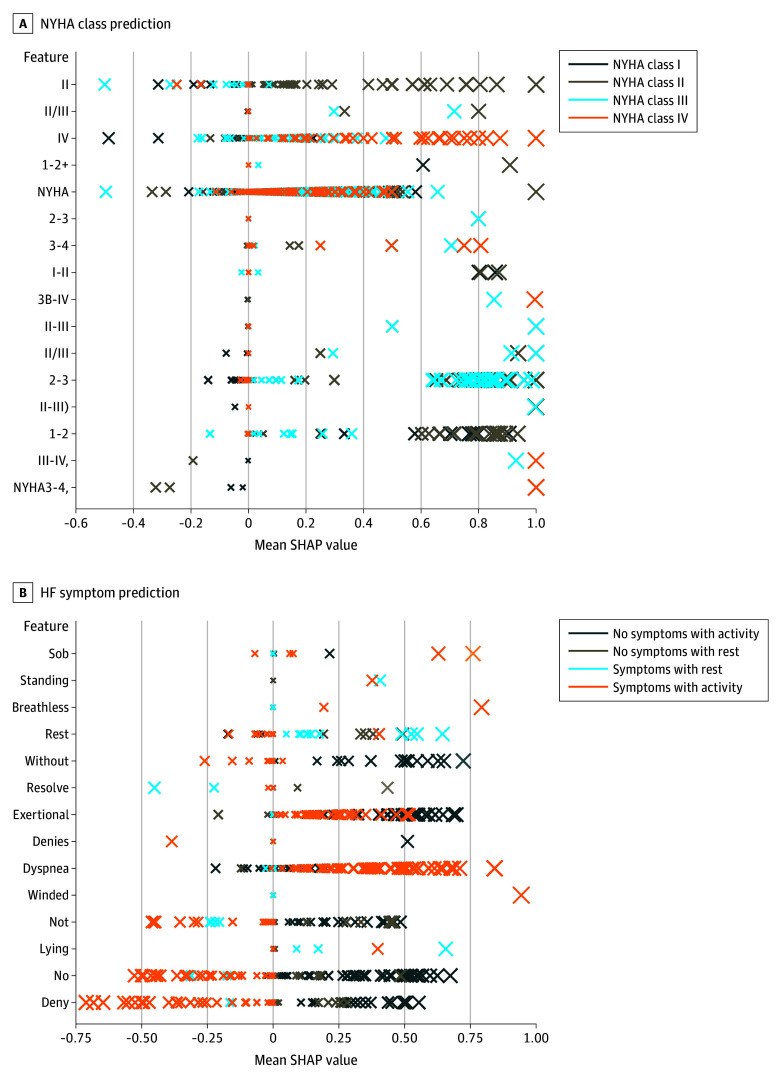
Top 15 Features by Mean Positive Shapley Additive Explanations (SHAP) Value Across New York Heart Association (NYHA) Class and Heart Failure (HF) Activity- and Rest-Related Symptom Models The y-axis lists individual features, and the x-axis represents the SHAP value indicating the feature’s impact on model predictions. Each “X” represents a clinical note, with the size of the “X” representing the weight of the token presented on the y-axis on model prediction. Higher SHAP values suggest greater importance of the feature in the model’s decision-making process.

SHAP-based interpretability analysis revealed key features NLP models leveraged for classifying the NYHA classes and the presence of HF symptoms (Figure 1). In the model for NYHA classification, designations such as *NYHA*, *Class*, and the corresponding Roman numerals *I*, *II*, *III*, and *IV* were the highest weighted features, consistent with the classification criteria detailed in our annotation guidelines. For the activity- and rest-related symptom model, clinical descriptors of symptoms, particularly *dyspnea* and *breathlessness*, alongside verbs correlating with physical exertion or movement, like *standing* and *exertional*, were the highest weighted features.

### Evaluation of NYHA Classification and Activity- and Rest-Related Symptoms Across Notes

**Figure 2.**
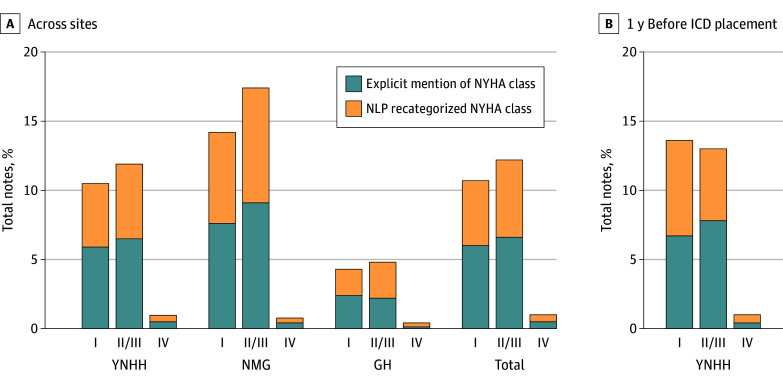
Frequency of New York Heart Association (NYHA) Class Mention and Recategorization by Activity-Related Symptoms GH indicates Greenwich Hospital; ICD, implantable cardioverter defibrillator; NLP, natural language processing; NMG, Northeast Medical Group; YNHH, Yale New Haven Hospital.

Across 182 308 outpatient notes at YNHH not used in either model development or validation, our NYHA-extraction algorithm identified explicit mentions of NYHA classes in 23 830 notes (13.1%), or approximately 1 in 8 notes. These were classified across the different classes, with 10 913 notes (6.0%) for class I, 12 034 notes (6.6%) for classes II or III, and 883 notes (0.5%) for class IV (Figure 2; eTable 8 in Supplement 1). The model that extracted descriptive mentions of activity- or rest-related HF symptoms could be used to additionally assign 19 730 notes (10.8%) to an NYHA class, including 8659 notes as class I (4.7%), 10 227 notes as classes II or III (5.6%), and 884 notes as class IV (0.5%) (Figure 2; eTable 8 in Supplement 1). Combining explicit mentions and NLP recategorizations resulted in a functional status classification in 43 560 notes (23.9%), representing an 83% increase in information capture compared with explicit mentions alone (eTable 8 in Supplement 1). We found that in the year before ICD implantation, among 5955 unique patient notes, only 887 notes (14.9%) had an explicit mention of NYHA class (Figure 2; eTable 9 in Supplement 1). The model that defined HF symptoms during activity or rest could be used to additionally assign 713 notes (12.7%) to an NYHA class (Figure 2; eTable 9 in Supplement 1).

### Subgroup Analysis

Our models demonstrated consistent performance across subgroups of race, sex, and EF categories, for both NYHA classification and detection of activity- or rest-related symptoms (eTable 10 and eTable 11 in Supplement 1). Across all subgroups, the micro-averaged AUROCs for the NYHA classification model ranged from 0.97 to 0.99 and the activity- and rest-related symptom model ranged from 0.93 to 0.94. Analysis by EF category showed that explicit NYHA mentions were more frequent in patients with reduced EF (20.2%) than those with preserved or mildly reduced EF (12.0%) (eTable 12 in Supplement 1).

## Discussion

In our multisite EHR diagnostic study, we developed and validated a novel deep learning approach to identify explicit NYHA functional status mentions from unstructured notes and classify activity-related symptoms into structured functional status groups when NYHA classes were not mentioned. Despite being a class I recommendation for patients with HF, only approximately 1 in 8 health encounters with patients with HF explicitly mentioned NYHA classes in the notes.^18^ Our model demonstrated robust performance in identifying these explicitly mentioned NYHA classes. Furthermore, the model could assign NYHA classes to descriptions of symptoms, increasing the capture of this information to nearly 1 in 4 notes. This substantial improvement in functional status documentation highlights both the current gaps in clinical practice as well as the potential of our approach to address them. Less than one-sixth of all notes (14.9%) within a year preceding ICD implantation explicitly mentioned NYHA classification. This finding highlights potential gaps in being able to monitor guideline adherence and decision-making processes for critical interventions, like ICD implantation, underscoring the value of our NLP approach in improving documentation and supporting clinical decision-making. The models exhibited excellent performance across internal and external validation populations, including a large academic hospital, a community-based multispecialty group practice, and a community teaching hospital, demonstrating generalizability across diverse health care settings. The interpretable inference confirmed that the models learned textual signatures consistent with features used by human abstractors. Our subgroup analyses demonstrated consistent performance of both the NYHA classification and models to identify activity- and rest-related symptoms across key demographic and clinical groups based on EF, suggesting that our approach can be scaled equitably across broad patient populations.

Our study offers a more efficient and scalable approach to extract functional status information from EHR compared with previous methods. Previous studies have used support vector machines and random forests with n-gram features to identify NYHA classes, but these methods relied on structured diagnosis codes and simplified text representations, potentially limiting their ability to capture the full context and nuance of functional status assessment in diverse clinical narratives.^20,21^ More recently, researchers have developed an ensemble method combining decision trees, random forests, and support vector machines for NYHA classification. This achieved high accuracy but relied heavily on structured data inputs, such as exercise capacity metrics and blood biomarkers, rather than unstructured text.^22^ In contrast, our deep learning NLP framework can process large volumes of unstructured data, identifying complex patterns and contextual information that may be missed by previous methods. This approach enables a more accurate and complete assessment of functional capacity across diverse clinical scenarios without manual feature engineering. Moreover, our model’s ability to both identify explicit NYHA mentions and infer functional status from symptom descriptions represents a more comprehensive solution for extracting this critical information from clinical notes.

Our automated approach has key implications for improving HF clinical documentation. First, our NLP models can facilitate quality measurement initiatives by providing a reliable means to track functional status assessments and their associations with clinical decision-making. This complements, rather than replaces, clinician assessment and documentation, offering a scalable solution to improve adherence to guideline-directed medical therapy. Second, the models can be integrated into clinical decision support systems, alerting clinicians when functional status assessments are due or when treatment plans may need adjustment based on a patient’s current functional capacity. This could also further increase the capture of this information in documentation via a clear identification of when this information has not been recorded. In addition, the high performance of our model in identifying NYHA class IV symptoms suggests a promising application in early detection of patients with advanced HF who are candidate for ICD. This capability could facilitate timely referrals for advanced therapies, such as left ventricular assist devices or heart transplantation. Finally, our approach can streamline patient identification for clinical trials by enabling the rapid screening of large patient cohorts based on their functional status, a key inclusion criterion for many HF studies.^23^ For instance, recent landmark studies have used the NYHA class as a key inclusion criterion.^24,25,26^ Our approach, which classifies twice as many encounters into NYHA classes as those explicitly mentioning the class information, could expedite recruitment in such trials, potentially accelerating the development of novel therapies.

Interestingly, our interpretability analysis revealed that the models not only learned to recognize explicit mentions of NYHA classes but also picked up on key phrases and descriptors associated with different levels of functional impairment. This suggests that the models can capture the underlying clinical approach used by clinicians when assessing a patient’s functional status, even in the absence of standardized terminology. This capability is particularly valuable, given the inherent variability in how functional status is documented across different clinicians and institutions.

### Limitations

Our study has some limitations. First, our NLP models’ performance may be influenced by the annotation guidelines and criteria used during model development. While we aimed to create a generalizable framework, institution-specific documentation practices or variations in clinical terminology could impact the models’ accuracy when applied to other settings. Nevertheless, the various practice sites share few clinicians, are geographically separated, and have practice patterns largely governed by local patient populations, suggesting the likely generalizability of the tool outside the tested hospitals and clinics. Second, our study relied on a single primary annotator; however, to mitigate potential biases, we implemented a quality control process from the outset of our study. Future work should consider using multiple annotators to enhance the robustness of the annotations further. Third, the complexity of HF symptoms and the potential for comorbid conditions to contribute to functional impairment may not be fully captured by our current models, particularly in more nuanced clinical scenarios. Future work should focus on refining the models to better account for these complexities and to incorporate additional clinical context when available. Additionally, the descriptions of class II (“mild symptoms and slight limitation during ordinary activity”) and class III (“marked limitation in activity due to symptoms, even during less-than-ordinary activity”) often overlap in clinical notes due to interphysician variability in documentation.^27^ This makes it challenging to reliably differentiate between the 2 classes from the notes alone. Additionally, we opted for scalability and model efficiency over model size and, therefore, chose to use a ClinicalBERT model over more recently described large language models. However, given the high performance of our model in detecting the condition, along with the ease of deploying a model that does not require a large graphical processing unit capacity, we believe the decision to use a lightweight model that is easy to deploy in practice is appropriate.

## Conclusions

In this diagnostic study, we developed and validated a deep-learning NLP approach to extract NYHA symptom class and activity- and rest-related HF symptoms from clinical notes. This scalable solution could enable tracking of patients receiving optimal care and enhance the automated identification of those eligible for clinical trials using existing clinical documentation.

## Notes

Supplement 1.
eMethods.
**eFigure 1.** Map of Yale New Haven Health System Internal and External Sites. **eFigure 2.** Development of Study Cohort Flowchart. **eFigure 3.** Study Overview **eFigure 4.** Algorithm for Natural Language Processing Based New York Health Association Class Recategorization **eFigure 5.** Distribution of Annotated Labels for New York Health Association Classification Model **eFigure 6.** Distribution of Annotated Labels for Description of Heart Failure Symptoms During Activity or Rest Model **eFigure 7.** Micro- and Macro-Averaged Area Under the Receiver Operating Characteristic Curve of Natural Language Processing Models in Characterizing Functional Status Labels **eTable 1.** Cardiovascular Outpatient Centers Affiliated With Yale New Haven Hospital, Northeast Medical Group, and Greenwich Hospital that Served as Validation Sites for the Study **eTable 2.** International Classification of Disease Diagnosis Codes for Identification of Patients With Heart Failure and Comorbidities **eTable 3.** Definitions and Pertinent Examples of Functional Status Labels **eTable 4.** Dictionary and CPT Codes for Identifying ICD Implantation **eTable 5.** Frequency of NYHA Classification in Manual Annotation of Outpatient Notes **eTable 6.** Distribution of Documented HF symptoms in Outpatient Notes at the 3 Validation Sites **eTable 7.** Performance Metrics of NLP Models in Classifying Each Functional Status Sublabel **eTable 8.** Postdeployment Assessment of Functional Status Across the Health System **eTable 9.** Postdeployment Assessment of Functional Status Across Patients at Yale New Haven Hospital for the Year Preceding Implantable Cardiovascular Defibrillator Procedure **eTable 10.** Performance Metrics of NYHA Class NLP Model Across Subgroups **eTable 11.** Performance Metrics of Symptom Association NLP Model Across Subgroups **eTable 12.** Postdeployment Analysis of NYHA Classification by Ejection Fraction Category
eReferences.

Supplement 2.
Data Sharing Statement
