# Supplementary material for: Natural Language Processing of Clinical Documentation to Assess Functional Status in Patients With Heart Failure
Source: JAMA Netw Open. 2024 Nov 7;7(11):e2443925. doi: 10.1001/jamanetworkopen.2024.43925 (PMC11544492; doi:10.1001/jamanetworkopen.2024.43925)
Supplement: Supplement 1. — eMethods. eFigure 1. Map of Yale New Haven Health System Internal and External Sites. eFigure 2. Development of Study Cohort Flowchart. eFigure 3. Study Overview eFigure 4. Algorithm for Natural Language Processing Based New York Health Association Class Recategorization eFigure 5. Distribution of Annotated Labels for New York Health Association Classification Model eFigure 6. Distribution of Annotated Labels for Description of Heart Failure Symptoms During Activity or Rest Model eFigure 7. Micro- and Macro-Averaged Area Under the Receiver Operating Characteristic Curve of Natural Language Processing Models in Characterizing Functional Status Labels eTable 1. Cardiovascular Outpatient Centers Affiliated With Yale New Haven Hospital, Northeast Medical Group, and Greenwich Hospital that Served as Validation Sites for the Study eTable 2. International Classification of Disease Diagnosis Codes for Identification of Patients With Heart Failure and Comorbidities eTable 3. Definitions and Pertinent Examples of Functional Status Labels eTable 4. Dictionary and CPT Codes for Identifying ICD Implantation eTable 5. Frequency of NYHA Classification in Manual Annotation of Outpatient Notes eTable 6. Distribution of Documented HF symptoms in Outpatient Notes at the 3 Validation Sites eTable 7. Performance Metrics of NLP Models in Classifying Each Functional Status Sublabel eTable 8. Postdeployment Assessment of Functional Status Across the Health System eTable 9. Postdeployment Assessment of Functional Status Across Patients at Yale New Haven Hospital for the Year Preceding Implantable Cardiovascular Defibrillator Procedure eTable 10. Performance Metrics of NYHA Class NLP Model Across Subgroups eTable 11. Performance Metrics of Symptom Association NLP Model Across Subgroups eTable 12. Postdeployment Analysis of NYHA Classification by Ejection Fraction Category eReferences. [file jamanetwopen-e2443925-s001.pdf]

Supplemental Online Content

Adejumo P, Thangaraj PM, Dhingra LS, et al. Natural language processing of clinical documentation to assess functional status in patients with heart failure. *JAMA Netw Open*. 2024;7(11):e2443925. doi:10.1001/jamanetworkopen.2024.43925

eMethods.
eFigure 1. Map of Yale New Haven Health System Internal and External Sites.
eFigure 2. Development of Study Cohort Flowchart.
eFigure 3. Study Overview
eFigure 4. Algorithm for Natural Language Processing Based New York Health Association Class Recategorization
eFigure 5. Distribution of Annotated Labels for New York Health Association Classification Model
eFigure 6. Distribution of Annotated Labels for Description of Heart Failure Symptoms During Activity or Rest Model
eFigure 7. Micro- and Macro-Averaged Area Under the Receiver Operating Characteristic Curve of Natural Language Processing Models in Characterizing Functional Status Labels
eTable 1. Cardiovascular Outpatient Centers Affiliated With Yale New Haven Hospital, Northeast Medical Group, and Greenwich Hospital that Served as Validation Sites for the Study
eTable 2. International Classification of Disease Diagnosis Codes for Identification of Patients With Heart Failure and Comorbidities
eTable 3. Definitions and Pertinent Examples of Functional Status Labels
eTable 4. Dictionary and CPT Codes for Identifying ICD Implantation
eTable 5. Frequency of NYHA Classification in Manual Annotation of Outpatient Notes
eTable 6. Distribution of Documented HF symptoms in Outpatient Notes at the 3 Validation Sites
eTable 7. Performance Metrics of NLP Models in Classifying Each Functional Status Sublabel
eTable 8. Postdeployment Assessment of Functional Status Across the Health System
eTable 9. Postdeployment Assessment of Functional Status Across Patients at Yale New Haven Hospital for the Year Preceding Implantable Cardiovascular Defibrillator Procedure
eTable 10. Performance Metrics of NYHA Class NLP Model Across Subgroups
eTable 11. Performance Metrics of Symptom Association NLP Model Across Subgroups
eTable 12. Postdeployment Analysis of NYHA Classification by Ejection Fraction Category
eReferences

This supplemental material has been provided by the authors to give readers additional information about their work.

© 2024 Adejumo P et al. *JAMA Network Open.*

eMethods

Software and Libraries

A Python-based computational environment was used for all analysis. The following libraries were employed:

- Pandas (version 1.5.3) for data processing
- Numpy (version 1.23.5) and SciPy (version 1.10.1) for numerical operations
- Scikit-learn (version 1.2.2) for machine learning tasks
- Prodigy (version 1.12) for corpus annotation
- SpaCy (version 3.6) for model development and refinement

We chose these libraries for their robustness, extensive documentation, and wide adoption in the medical NLP community. Pandas and Numpy provided efficient data manipulation capabilities, while Scikit-learn offered a comprehensive suite of machine learning tools. Prodigy facilitated our annotation process, and SpaCy provided specialized NLP functionalities for clinical text.

Data Pre-processing

We sectioned all notes to include only the history of presenting illness and the assessment *and* plan. This focused the analysis on the most relevant sections for assessing functional status and NYHA classification. We leveraged MedSpaCy's clinical sectionizer to tag and remove other sections of the outpatient medical note, including the past medical history, labs, studies, allergies, physical exam, medications, family history, and imaging.[1] The review of systems section was also excluded from our analysis. While this section can contain relevant symptom information, it often lacks the detailed activity or rest-related context necessary for accurate NYHA classification, which is more reliably captured in the history of presenting illness and assessment/plan sections.

In the selected components of the note, we pursued a sentence-level annotation process to identify sentences containing information relevant to the patient's functional status and NYHA classification. For this, we split the notes into individual sentences using a clinically trained transformer-based sentence tokenizer. *Each sentence was then labeled as either containing functional status information or not. The annotator was presented with one sentence at a time, in random order, to reduce potential bias from the surrounding context. This approach allowed for a focused assessment of each sentence's relevance to functional status, while still maintaining the ability to reconstruct the full note context later in the process.* The labeled sentences were later reassembled to reconstruct the context of each note, enabling note-level classification based on the aggregated sentence-level labels. This step was critical for ensuring the integrity of the note-level classification.

Model Development

We chose to finetune a ClinicalBERT-based model, a lightweight variant of the Bidirectional Encoder Representations from Transformers (BERT) architecture that has been specifically pre-trained on a large corpus of clinical text. This approach leverages transfer learning, utilizing the model's pre-existing training on clinical language. We used several key software packages for data preprocessing and model development, including MedSpaCy for clinical text processing and Hugging Face Transformers for implementing the BERT model.

Interpretability Analysis

© 2024 Adejumo P et al. *JAMA Network Open.*

For our interpretability analysis, we applied an adapted version of the SHapley Additive exPlanations (SHAP) method specifically designed for transformer-based classifiers like BERT. This adapted SHAP method quantifies the impact of each feature on the NLP models' outputs while accounting for the complex feature interactions in transformer architectures.

Statistical Analysis

Micro-averaged metrics were chosen as the primary focus of our analysis, as they provide a comprehensive assessment of the model's performance across individual classes, giving equal weight to each classification decision. This approach is particularly suitable for our study, as it aligns with our objective of evaluating the overall performance of the NLP models in identifying functional status information across the entire dataset. However, it is important to note that micro-averaging can potentially overemphasize the performance of larger classes. Macro-averaged metrics, which treat all classes equally, regardless of their frequency, were also reported to provide additional insights into the model's performance on individual classes. This approach is particularly useful for understanding the model's performance on less common NYHA classes, as it gives equal importance to each class in the final metric. While macro-averaging provides a balanced view across classes, it may not fully reflect the model's performance in real-world scenarios where class distributions are imbalanced.

Annotating Guidelines

The primary objective of our annotation task is to systematically classify notes and sentences based on HF functional status. At the note-level, patient notes are organized into categories of NYHA Class I, II/III, IV, or cases with no NYHA Class mentioned. This categorization helps establish the severity of heart failure in each patient, providing context for the functional status assessment. We combined NYHA Classes II and III due to their combination in guidelines for treatment decisions and the significant inter-physician variability in documentation.[2–4] This approach aligns with common practices in heart failure clinical trials, which frequently use combined NYHA II-III categories for eligibility criteria.[5–8] Each note is tokenized into sentences and initially labeled as either pertaining to 'functional status' or 'not functional status'. Subsequently, those identified as 'functional status' sentences undergo a more nuanced subclassification. These subclasses are designed to capture various aspects of a patient's functional status and include categories such as 'symptoms with activity', 'no symptoms with activity', 'symptoms with rest', 'no symptoms with rest', 'activity ability', and 'activity limitation'.

In the process of annotating outpatient medical notes, it is important for annotators to focus solely on the content within each sentence, without drawing upon the surrounding context. This approach ensures a standardized and objective analysis of each sentence, aligning with our goal of identifying HF functional status indicators. Annotators must operate under the assumption that any mention of symptoms or limitations in a patient's functional status is related to HF, except in cases where other specific causes are explicitly stated. For instance, a sentence stating "symptoms limit the patient's ability to walk up a flight of stairs" should be interpreted as HF-related unless other conditions are clearly identified.

Additionally, annotators are encouraged to consider the writer's intent and the clinical relevance of each sentence. Understanding why a healthcare provider chose to include specific information is crucial. For example, a sentence detailing a patient's daily activities or limitations provides valuable insight into their functional status and should be considered relevant to HF

© 2024 Adejumo P et al. *JAMA Network Open.*

unless stated otherwise. This perspective aids in capturing the nuances of clinical communication, enhancing the quality and applicability of the annotated data. Annotators must strictly adhere to the provided definitions and guidelines, ensuring that each sentence is classified accurately and consistently.

Quality Control Process

To mitigate potential bias from using a single primary annotator, we implemented a rigorous quality control process. A panel of two secondary clinical experts regularly evaluated a random sample of annotated notes. Any discrepancies or uncertainties identified during these checks were discussed and resolved by the expert panel, with the annotation guidelines updated if necessary. This process helped ensure consistency in the annotations and allowed for ongoing refinement of the annotation guidelines throughout the study.

NYHA Class Manual Annotation

The NYHA Classification labels are based on explicit mention of active current NYHA class categorization by a documented physician.

Qualifying Criteria:

- Sentences where a healthcare provider explicitly mentions active NYHA classification.
- Sentences describing symptoms clearly aligning with a specific NYHA class, even if the class is not explicitly mentioned.
- Direct statements about the patient's physical ability or limitations correlating with a specific NYHA class.

Non-Qualifying Criteria:

- Sentences that make broad statements about the patient’s health without specific reference to NYHA class or related symptoms.
- Descriptions of symptoms or limitations clearly attributed to conditions other than heart failure.
- Past or future medical history that does not reflect the current NYHA class.

Notes and Edge Cases:

- In cases where symptoms could be attributed to multiple classes, use the most restrictive class (highest number) that the symptoms could indicate.
- When a sentence contains both qualifying and non-qualifying information, prioritize the information that aligns with the NYHA classification.

Symptom Association Manual Annotation

The category of ‘Symptom Association’ encompasses sentences that explicitly describe a patient's physical abilities, limitations, or symptoms as they relate to daily activities and rest, particularly in the context of HF. To qualify as a ‘Symptom Association' sentence, the text must provide clear information about the patient's capability or incapacity to perform tasks, or the presence of symptoms during these activities or at rest.

Qualifying Criteria:

© 2024 Adejumo P et al. *JAMA Network Open.*

- Sentences that mention symptoms (like dyspnea, fatigue) occurring during physical activities.
- Sentences indicating symptoms experienced by the patient at rest.

Non-Qualifying Criteria:

- General health statements that do not specifically address the patient’s current functional status.
- References to medical treatments or medications without explicit mention of their impact on functional status.
- Historical information about the patient’s abilities or symptoms not relevant to their current condition.

Notes and Edge Cases:

In cases where the classification of a sentence is ambiguous or unclear, annotators should adhere to the following guidelines:

- When a sentence implicitly suggests a change in functional status without direct mention (e.g., "Patient now requires assistance for walking"), classify it as 'Functional Status'. The underlying assumption is that the change is significant enough to be noted in the patient's medical record.
- If a sentence describes symptoms without explicitly attributing them to HF but no other causes are mentioned (e.g., "Experiences shortness of breath while climbing stairs"), assume the symptoms are HF-related and classify accordingly.
- Distinguish between historical anecdotes and current functional status. Focus on the current state; historical references are not to be classified as 'Functional Status' unless they directly impact or explain the current condition.
- Broad or general statements about the patient’s health (e.g., "Patient is doing well") should not be classified under 'Functional Status' unless they include specific information about physical abilities or symptoms

© 2024 Adejumo P et al. *JAMA Network Open.*

eFigure 1. Map of Yale New Haven Health System Internal and External Sites. Abbreviations: GH, Greenwich Hospital; NMG, Northeast Medical Group; YNHH, Yale New Haven Hospital

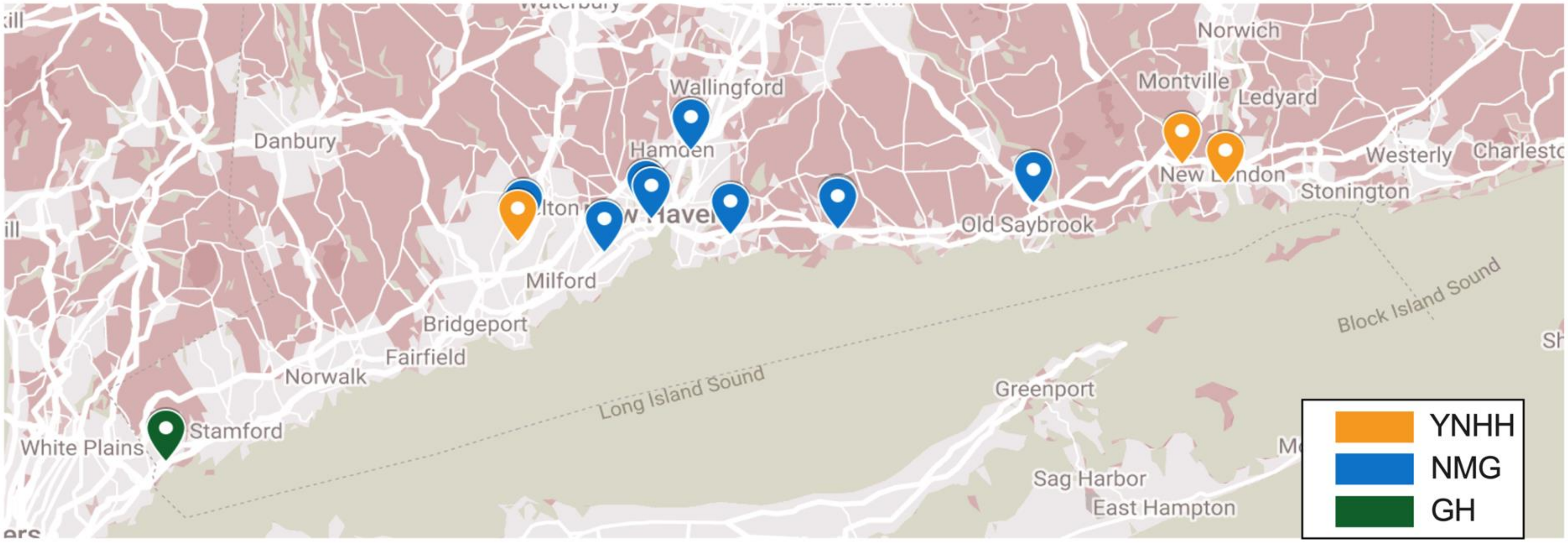

© 2024 Adejumo P et al. *JAMA Network Open.*

eFigure 2. Development of Study Cohort Flowchart. Abbreviations: ICD, International Classification of Disease.

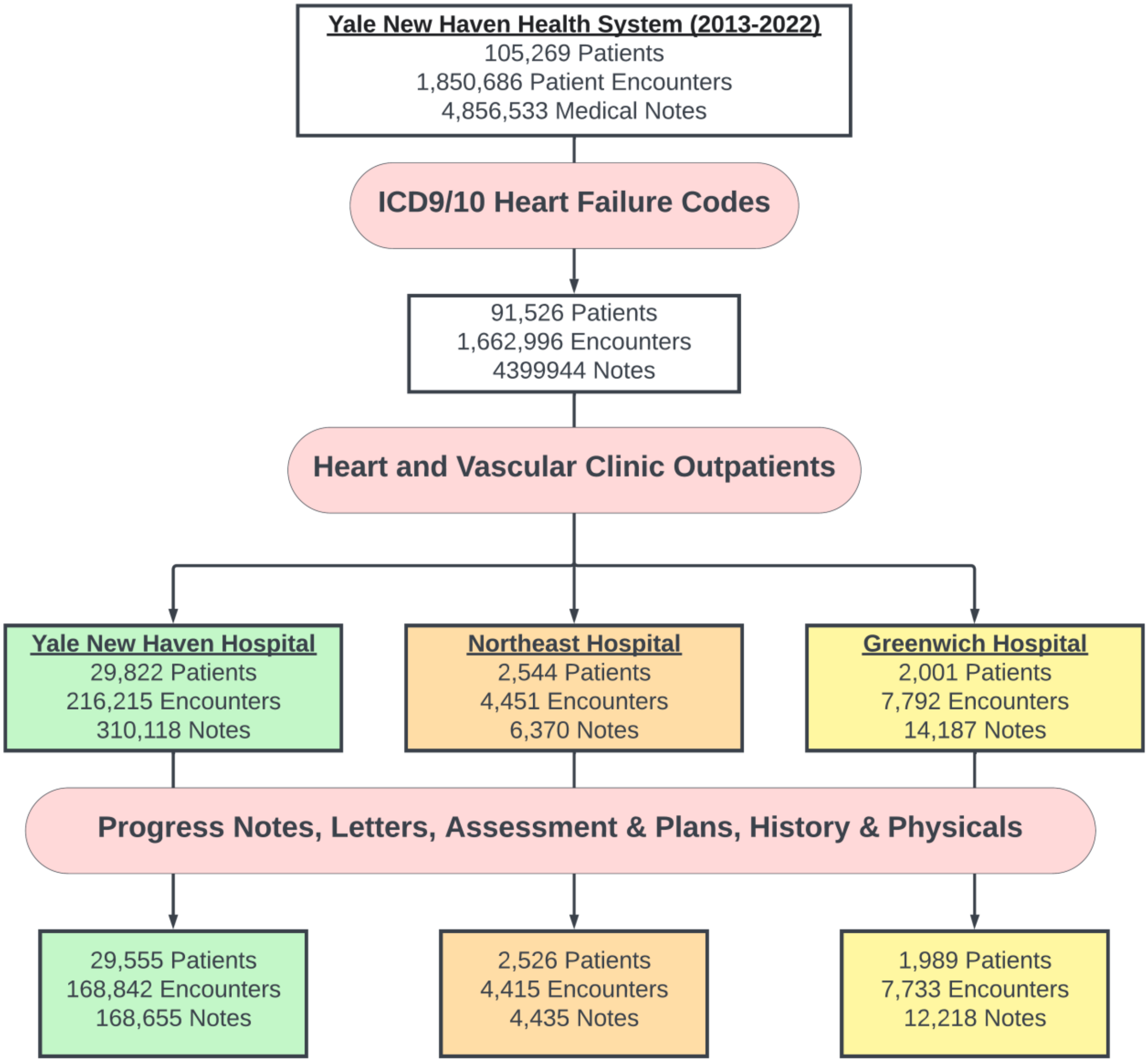

© 2024 Adejumo P et al. *JAMA Network Open.*

eFigure 3. Study Overview including (1) the annotation scheme for annotation of New York Heart Association class and functional activity and (2) the model development, internal and external validation, and deployment strategies. Abbreviations: GH, Greenwich Hospital; NMG, Northeast Medical Group; NYHA, New York Heart Association; Val, Validation; YNHH, Yale New Haven Hospital.

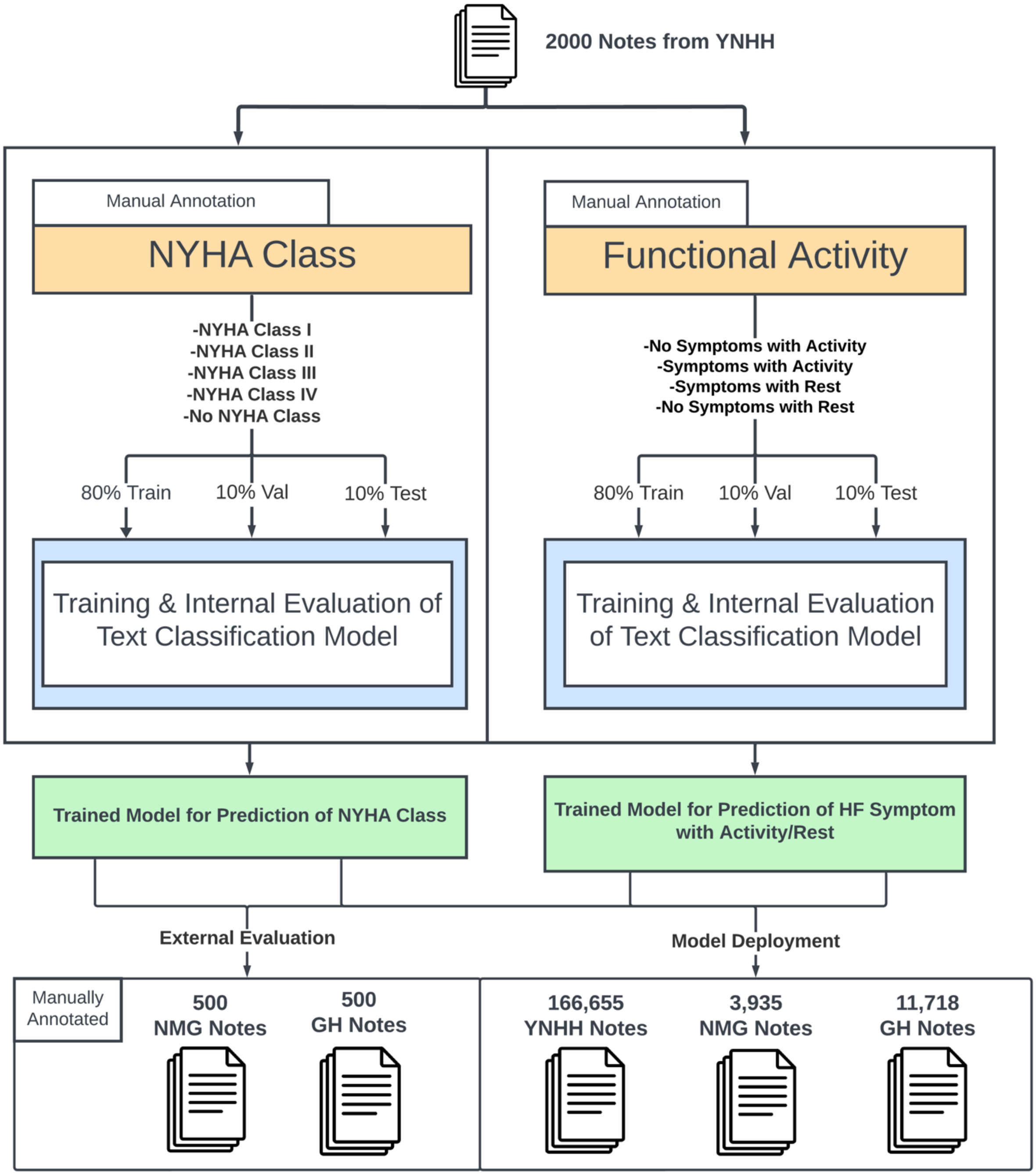

© 2024 Adejumo P et al. *JAMA Network Open.*

eFigure 4. Algorithm for Natural Language Processing Based New York Health Association Class Recategorization. Abbreviations: HF, Heart Failure; NYHA, New York Health Association

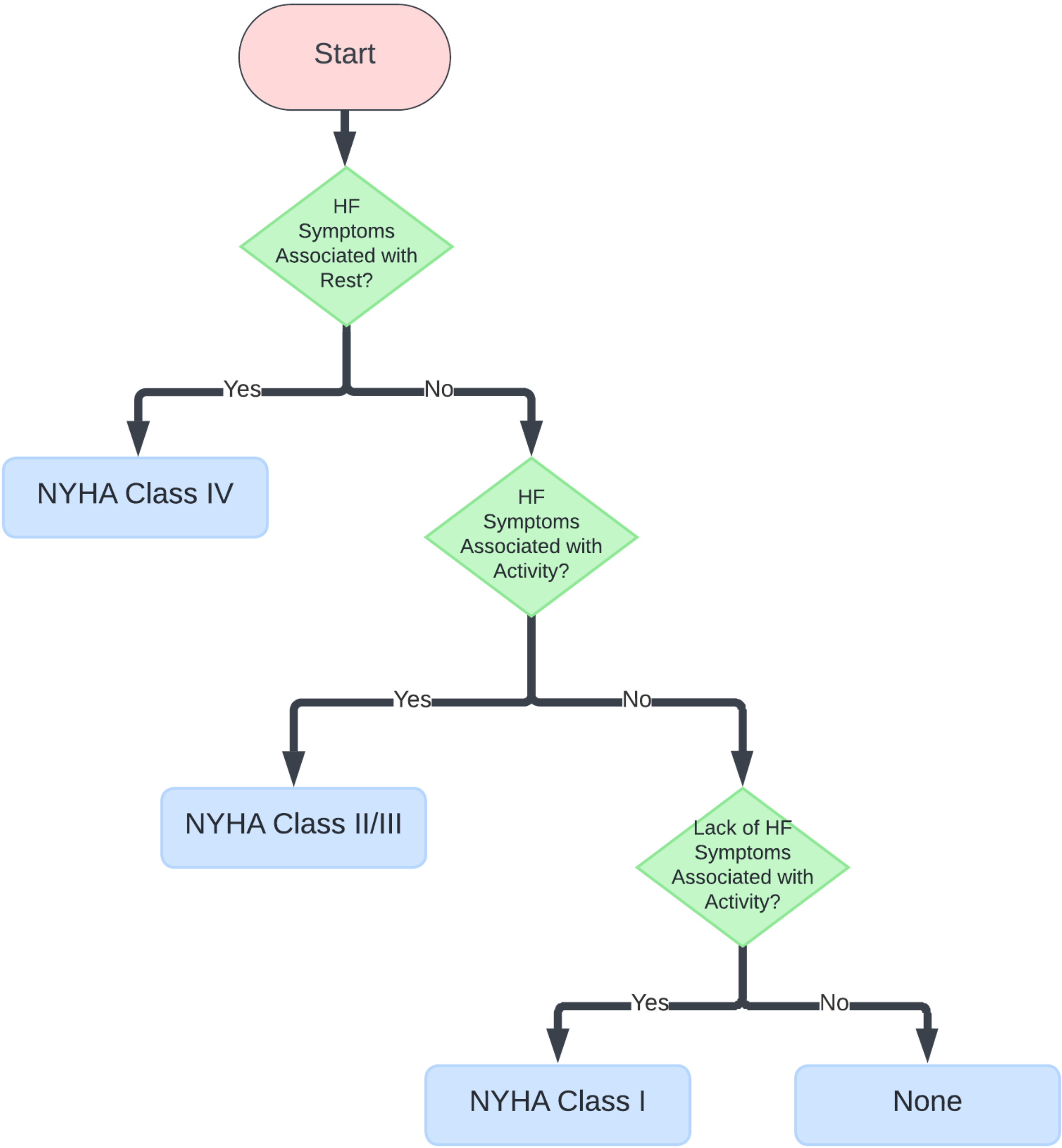

© 2024 Adejumo P et al. *JAMA Network Open.*

eFigure 5. Distribution of Annotated Labels for New York Health Association Classification Model. Abbreviations: GH, Greenwich Hospital; NMG, Northeast Medical Group; NYHA, New York Heart Association; YNH, Yale New Haven Hospital.

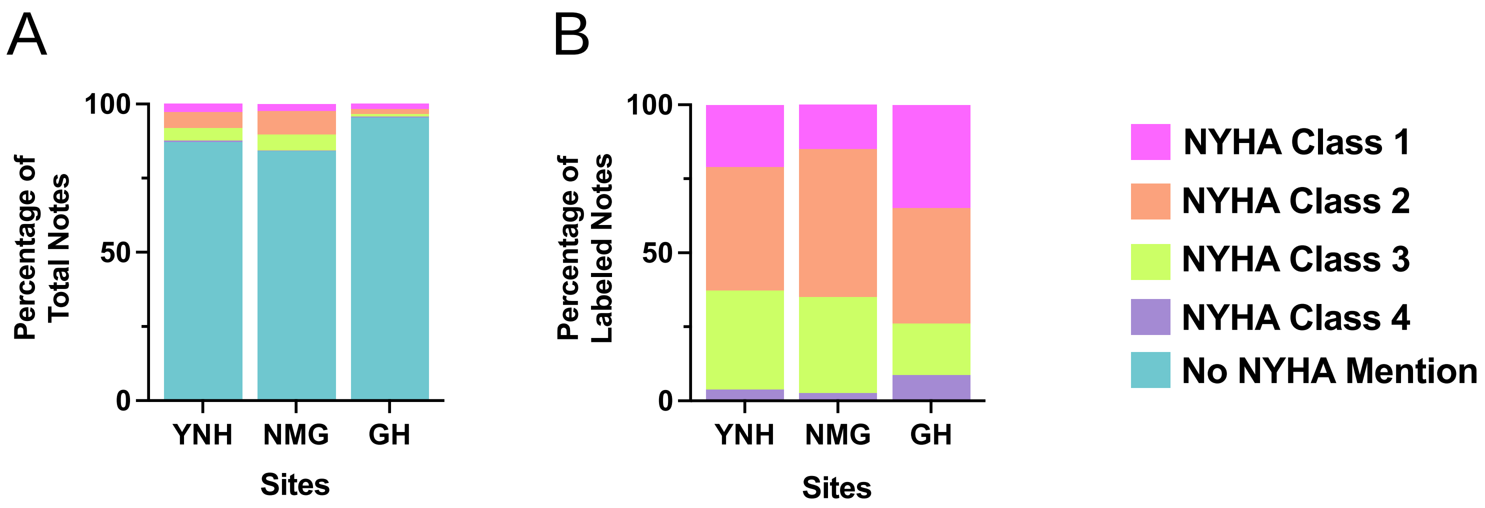

© 2024 Adejumo P et al. *JAMA Network Open.*

eFigure 6. Distribution of Annotated Labels for Description of Heart Failure Symptoms During Activity or Rest Model.
Abbreviations: GH, Greenwich Hospital; NMG, Northeast Medical Group; YNH, Yale New Haven Hospital.

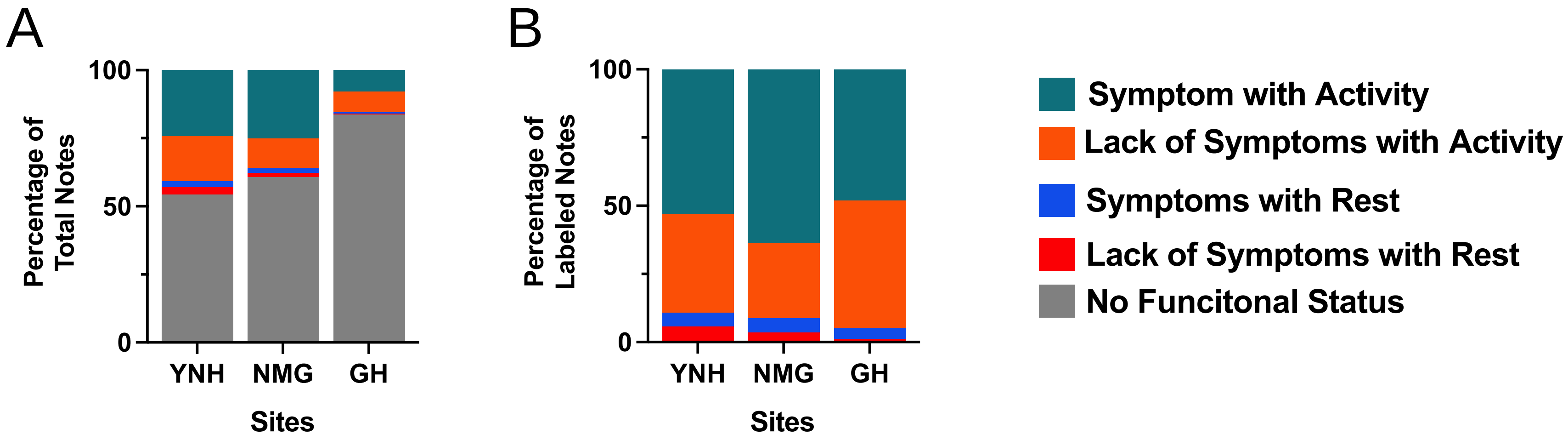

© 2024 Adejumo P et al. *JAMA Network Open.*

eFigure 7. Micro- and Macro-Averaged Area Under the Receiver Operating Characteristic Curve of Natural Language Processing Models in Characterizing Functional Status Labels. Abbreviations: AUROC, area under the receiver operating characteristic curve; GH, Greenwich Hospital; NMG, Northeast Medical Group; NYHA, New York Heart Association; YNHH, Yale New Haven Hospital.

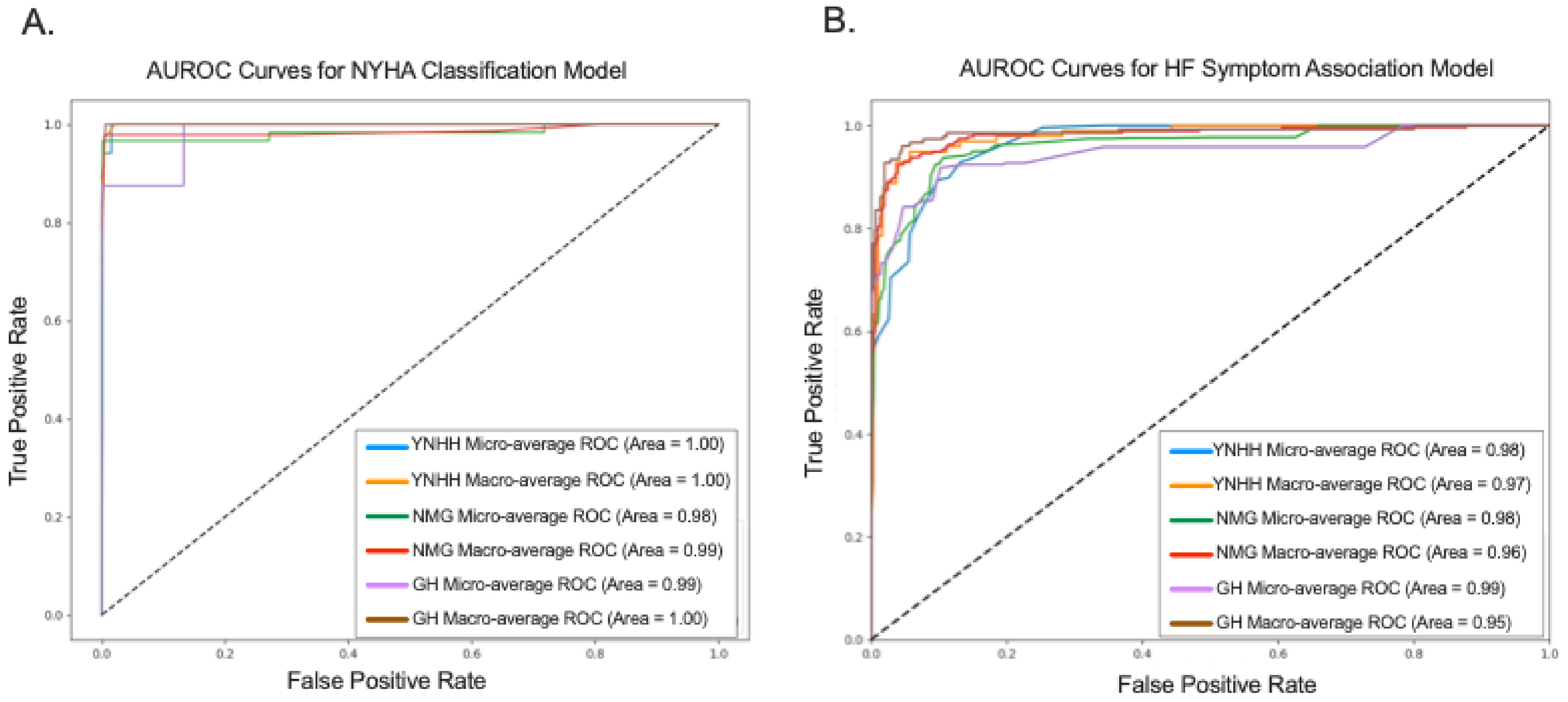

© 2024 Adejumo P et al. *JAMA Network Open.*

eTable 1. **Cardiovascular outpatient centers affiliated with Yale New Haven Hospital, Northeast Medical Group, and Greenwich Hospital that served as validation sites for the study.**

Validation Site	Cardiovascular Outpatient Centers
Yale New Haven Hospital	Heart & Vascular Clinic North Haven, Heart & Vascular Clinic Branford, Heart & Vascular Clinic ORANGE', Heart & Vascular Clinic Guilford, Heart & Vascular Clinic Westbrook, Heart & Vascular Clinic Transition Care, Branford Ambulatory Cardiology Service, Yale New Haven Cardiac Rehab Center, Yale Medicine New Haven Sherman Avenue, Yale New Haven Congestive Heart Failure Clinic, New Haven Ambulatory Cardiology Service, Guilford Ambulatory Cardiology Service, Yale New Haven Prevention and Fellows Clinic, Old Saybrook Ambulatory Cardiology
Northeast Medical Group	Lawrence & Memorial Cardiology Waterford, Shelton Cardiology Group, New London Cardiology Group, Norwich Cardiology Group
Greenwich Hospital	Greenwich Hospital Outpatient Cardiology

© 2024 Adejumo P et al. *JAMA Network Open.*

eTable 2. International Classification of Disease Diagnosis Codes for Identification of Patients with Heart Failure and Comorbidities.

Condition	ICD-9 Codes	ICD-10 Codes
Heart Failure	402.x, 404.x, 428.0, 428.1, 428.2, 428.20, 428.21, 428.22, 428.23, 428.3, 428.30, 428.31, 428.32, 428.33, 428.4, 428.40, 428.41, 428.42, 428.43, 428.9	I50.1, I50.2, I50.20, I50.21, I50.22, I50.23, I50.3, I50.30, I50.31, I50.32, I50.33, I50.4, I50.40, I50.41, I50.42, I50.43, I50.8, I50.81, I50.810, I50.811, I50.812, I50.813, I50.814, I50.82, I50.83, I50.84, I50.89, I50.9
Acute Myocardial Infarction	410.x	I21.x, I22.x
Cardiomyopathy	425.x	I42.x, I43.x
Hypertension	401.x, 402.x, 403.x, 404.x, 405.x	I10, I11.x, I12.x, I13.x, I15.x
Diabetes	250.x	E08.x, E09.x, E10.x, E11.x, E13.x
Chronic Kidney Disease	585.x, 586	N18.x, N19

© 2024 Adejumo P et al. *JAMA Network Open.*

eTable 3. Definitions and Pertinent Examples of Functional Status Labels. Abbreviations: HF, Heart Failure; NYHA, New York Heart Association.

**Label**	**Definition**	**Pertinent Example**
**NYHA Class Model**		
NYHA Class I	A widely recognized system for categorizing heart failure severity based on symptoms and limitations in physical activity. It ranges from Class I (no symptoms) to Class IV (severe limitations).	"Patient presents with NYHA Class II symptoms, experiencing slight limitation during ordinary activity."
NYHA Class II		
NYHA Class III		
NYHA Class IV		
**HF Symptom Association Model**		
HF Symptoms Associated with Activity	Descriptions of heart failure symptoms that become evident or worsen during physical exertion.	"During moderate exercise, the patient reports shortness of breath and palpitations."
Lack of HF Symptoms Associated with Activity	Refers to the absence of heart failure symptoms during physical activity, indicating a lesser severity of HF.	"Patient shows no signs of fatigue or dyspnea during routine physical activities."
HF Symptoms Associated with Rest	Descriptions of heart failure symptoms that persist or are present even when the patient is at rest.	"Patient experiences fatigue and dyspnea, even while at rest."
Lack of HF Symptoms Associated with Rest	Indicates the absence of heart failure symptoms while the patient is at rest, suggesting a controlled or mild form of HF.	"Patient reports feeling comfortable and symptom-free while resting."

© 2024 Adejumo P et al. *JAMA Network Open.*

eTable 4. Dictionary and CPT Codes for Identifying ICD Implantation. Abbreviations: CPT, Current Procedural Terminology; ICD, Implantable Cardiovascular Device

Procedure/Medication	Dictionary/CPT Codes
ICD Implantation	'Insert of Defib Gen into Chest Subcu/Fascia, Open Approach', 'Insertion of Defib Lead into R Ventricle, Perc Approach', 'Insertion of Defibrillator Lead into R Atrium, Perc Approach', 'Insert Card Rsync Defib Puls Gen in Chest Subcu/Fascia, Open', 'Insertion of Defibrillator Lead into Cor Vein, Perc Approach', 'IMPLT/REPL CARDDEFIB TOT', 'IMPL CRT DEFIBRILLAT SYS', 'IMP/REP CRT DEFIB GENAT', 'Insertion of Defib Lead into L Ventricle, Perc Approach', 'Insertion of Defib Lead into Pericardium, Open Approach', 'Insertion of Subcutaneous Defibrillator Lead into Chest Subcutaneous Tissue and Fascia, Open Approach', 'Insertion of Subcutaneous Defibrillator Lead into Chest Subcutaneous Tissue and Fascia, Percutaneous Approach', 'Insert Card Rsync Defib Puls Gen in Chest Subcu/Fascia, Perc', 'Insert of Defib Gen into Chest Subcu/Fascia, Perc Approach', 'Insertion of Defib Lead into R Ventricle, Open Approach', 'Insertion of Defib Lead into L Ventricle, Open Approach', 'IMPLT CARDIODEFIB LEADS', 'Insertion of Defib Lead into Pericardium, Perc Approach', 'Insertion of Defib Gen into Abd Subcu/Fascia, Open Approach', 'Insert Card Rsync Defib Puls Gen in Abd Subcu/Fascia, Open', 'Insertion of Defibrillator Lead into L Atrium, Open Approach', 'Insertion of Defibrillator Lead into L Atrium, Perc Approach', 'Insertion of Defibrillator Lead into R Atrium, Open Approach', 'Insert Card Rsync Defib Puls Gen in Abd Subcu/Fascia, Perc', 'Insertion of Defibrillator Lead into Cor Vein, Open Approach', 'Insertion of Defib Lead into R Atrium, Perc Endo Approach', 'IMPLT CARDIODEFIB GENATR', 'Insertion of Defib Lead into L Ventricle, Perc Endo Approach', 'Insertion of Defib Lead into L Atrium, Perc Endo Approach', 'Insertion of Defib Lead into R Ventricle, Perc Endo Approach', 'Insertion of Defib Lead into Cor Vein, Perc Endo Approach', '33216', '33217', '33225', '33230', '33231', '33240', '33249', '33270', '33271', 'C7537', 'C7538', 'C7539', 'G0448'

© 2024 Adejumo P et al. *JAMA Network Open.*

eTable 5. Frequency of NYHA Classification in Manual Annotation of Outpatient Notes. Abbreviations: NYHA, New York Heart Association

		**NYHA Class I**	**NYHA Class II**	**NYHA Class III**	**NYHA Class IV**	**Any NYHA Class**	**None**
**Yale New Haven Hospital**	Notes (n = 2,000)	57 (2.9%)	118 (5.9%)	86 (4.3%)	10 (0.5%)	271 (13.6%)	1729 (86.4%)
**Northeast Medical Group**	Notes (n = 500)	12 (2.4%)	40 (8.0%)	26 (5.2%)	2 (0.4%)	80 (16.0%)	420 (84.0%)
**Greenwich Hospital**	Notes (n = 500)	8 (1.6%)	9 (1.8%)	4 (0.8%)	2 (0.4%)	23 (4.6%)	477 (95.4%)
**Total**	Notes (n = 3,000)	77 (2.6%)	167 (5.6%)	116 (3.9%)	14 (0.5%)	374 (12.4%)	2626 (87.5%)

© 2024 Adejumo P et al. *JAMA Network Open.*

eTable 6. Distribution of Documented HF symptoms in Outpatient Notes at the Three Validation Sites.

		Symptoms with Activity	Lack of Symptoms with Activity	Symptoms with Rest	Lack of Symptoms with Rest	Any Description of Symptoms	None
**Yale New Haven Hospital**	Notes (n = 2,000)	486 (24.3%)	329 (16.5%)	45 (2.3%)	53 (2.7%)	913 (45.7%)	1087 (54.4%)
**Northeast Medical Group**	Notes (n = 500)	125 (25.0%)	54 (10.8%)	10 (2.0%)	7 (1.4%)	196 (39.2%)	304 (60.8%)
**Greenwich Hospital**	Notes (n = 500)	39 (7.8%)	38 (7.6%)	3 (0.6%)	1 (0.2%)	81 (16.2%)	419 (83.8%)
**Total**	Notes (n = 3,000)	650 (21.7%)	421 (14.0%)	58 (1.9%)	61 (2.0%)	1190 (39.7%)	1810 (60.3%)

© 2024 Adejumo P et al. *JAMA Network Open.*

eTable 7. Performance Metrics of NLP Models in Classifying Each Functional Status Sub-Label. Abbreviations: AUROC, Area under the receiver operating characteristic curve; CI, Confidence Interval; NYHA, New York Heart Association.

	**Accuracy (95% CI)**	**Precision (95% CI)**	**Recall (95% CI)**	**Specificity (95% CI)**	**AUROC (95% CI)**	**F_1-score (95% CI)**
**NYHA Class I**	0.97 (0.96-0.98)	0.22 (0.19-0.24)	1.00 (1.00-1.00)	0.97 (0.96-0.98)	0.98 (0.93-1.00)	0.36 (0.33-0.38)
**NYHA Class II**	0.98 (0.97-0.99)	0.64 (0.61-0.67)	0.97 (0.97-0.98)	0.98 (0.97-0.99)	0.98 (0.95-1.00)	0.77 (0.75-0.80)
**NYHA Class III**	0.98 (0.97-0.99)	0.57 (0.54-0.60)	0.94 (0.92-0.95)	0.98 (0.97-0.99)	0.96 (0.91-1.00)	0.71 (0.68-0.73)
**NYHA Class IV**	1.00 (1.00-1.00)	1.00 (1.00-1.00)	1.00 (1.00-1.00)	1.00 (1.00-1.00)	1.00 (1.00-1.00)	1.00 (1.00-1.00)
**Symptoms with Activity**	0.93 (0.91-0.95)	0.93 (0.91-0.95)	0.96 (0.95-0.98)	0.88 (0.85-0.90)	0.98 (0.96-0.99)	0.95 (0.93-0.97)
**Lack of Symptoms with Activity**	0.96 (0.94-0.97)	0.87 (0.84-0.90)	0.98 (0.96-0.99)	0.95 (0.93-0.97)	0.99 (0.99-1.00)	0.92 (0.90-0.94)
**Symptoms with Rest**	0.95 (0.93-0.97)	0.72 (0.68-0.76)	0.49 (0.44-0.53)	0.99 (0.97-1.00)	0.94 (0.92-0.96)	0.94 (0.92-0.96)
**Lack of Symptoms with Rest**	0.98 (0.97-0.99)	0.93 (0.91-0.95)	0.57 (0.52-0.61)	1.00 (0.99-1.00)	0.92 (0.90-0.95)	0.70 (0.66-0.74)

© 2024 Adejumo P et al. *JAMA Network Open.*

eTable 8. Post-Deployment Assessment of Functional Status Across the Health System.
Abbreviations: NYHA, New York Heart Association.

		Explicit NYHA Class Mention	Recategorized NYHA Class	Combined NYHA Class after Recategorization
Yale New Haven Hospital (n = 166,655)	NYHA Class I	9,878 (5.9%)	7,760 (4.6%)	17,638 (10.5%)
	NYHA Class II/III	10,824 (6.5%)	9,094 (5.4%)	19,918 (11.9%)
	NYHA Class IV	826 (0.5%)	788 (4.7%)	1,614 (1.0%)
	Subtotal	21,528 (12.9%)	17,642 (10.5%)	39,170 (23.5%)
Northeast Medical Group (n = 3,395)	NYHA Class I	929 (7.6%)	812 (6.6%)	1,741 (14.2%)
	NYHA Class II/III	1,112 (9.1%)	1,019 (8.3%)	2,131 (17.4%)
	NYHA Class IV	51 (0.4%)	43 (0.3%)	94 (0.7%)
	Subtotal	2,092 (17.1%)	1,874 (15.2%)	3,966 (32.3%)
Greenwich Hospital (n = 11,718)	NYHA Class I	106 (2.4%)	87 (1.9%)	193 (4.3%)
	NYHA Class II/III	98 (2.2%)	114 (2.6%)	212 (4.8%)
	NYHA Class IV	6 (0.1%)	13 (0.2%)	19 (0.3%)
	Subtotal	210 (4.7%)	214 (4.7%)	424 (9.4%)
All Sites Combined (n = 182,308)	NYHA Class I	10,913 (6.0%)	8,659 (4.7%)	19,572 (10.7%)
	NYHA Class II/III	12,034 (6.6%)	10,227 (5.6%)	22,261 (12.2%)
	NYHA Class IV	883 (0.5%)	844 (0.5%)	1,727 (0.9%)
	Total	23,830 (13.1%)	19,730 (10.8%)	43,560 (23.9%)

© 2024 Adejumo P et al. *JAMA Network Open.*

eTable 9. Post-Deployment Assessment of Functional Status Across Patients at Yale New Haven Hospital for the Year Preceding Implantable Cardiovascular Defibrillator Procedure. Abbreviations: NYHA, New York Heart Association.

		Explicit NYHA Class Mention	Recategorized NYHA Class	Combined NYHA Class after Recategorization
Yale New Haven Hospital (n = 5,955)	NYHA Class I	402 (6.7%)	388 (6.9%)	790 (13.6%)
	NYHA Class II/III	463 (7.8%)	291 (5.2%)	754 (13.0%)
	NYHA Class IV	22 (0.4%)	34 (0.6%)	56 (1.0%)
	Total	887 (14.9%)	713 (12.7%)	1,600 (27.6%)

© 2024 Adejumo P et al. *JAMA Network Open.*

eTable 10. Performance Metrics of NYHA Class NLP Model Across Subgroups. Abbreviations: AUROC, Area under the receiver operating characteristic curve; AUPRC, Area under the precision-recall curves; CI, Confidence Interval; NYHA, New York Heart Association.

Subgroup	Average	Accuracy (95% CI)	Precision (95% CI)	Recall (95% CI)	Specificity (95% CI)	AUROC (95% CI)	AUPRC (95% CI)	F1-Score (95% CI)
**Race**								
**White**	**Micro-average**	0.98 (0.96-1.00)	0.98 (0.96-1.00)	0.98 (0.96-1.00)	0.96 (0.96-1.00)	0.99 (0.98-1.00)	0.88 (0.84-0.93)	0.69 (0.63-0.76)
	**Macro-average**	0.98 (0.96-1.00)	0.58 (0.53-0.63)	1.00 (1.00-1.00)	0.98 (0.96-1.00)	0.99 (0.93-1.00)	0.58 (0.53-0.63)	0.69 (0.65-0.74)
**Black**	**Micro-average**	0.97 (0.95-0.99)	0.97 (0.95-0.99)	0.97 (0.95-0.99)	0.97 (0.95-0.99)	0.98 (0.96-1.00)	0.86 (0.81-0.91)	0.68 (0.62-0.74)
	**Macro-average**	0.97 (0.95-0.99)	0.57 (0.52-0.62)	0.99 (0.98-1.00)	0.97 (0.95-0.99)	0.98 (0.92-1.00)	0.57 (0.52-0.62)	0.68 (0.64-0.73)
**Asian**	**Micro-average**	0.98 (0.96-1.00)	0.98 (0.96-1.00)	0.98 (0.96-1.00)	0.97 (0.96-0.99)	0.99 (0.97-1.00)	0.87 (0.82-0.92)	0.69 (0.62-0.76)
	**Macro-average**	0.98 (0.96-1.00)	0.58 (0.52-0.64)	0.99 (0.98-1.00)	0.98 (0.96-1.00)	0.98 (0.92-1.00)	0.58 (0.52-0.64)	0.69 (0.64-0.74)
**Other**	**Micro-average**	0.97 (0.95-0.99)	0.97 (0.95-0.99)	0.97 (0.95-0.99)	0.96 (0.94-0.98)	0.98 (0.96-1.00)	0.85 (0.80-0.90)	0.67 (0.61-0.73)
	**Macro-average**	0.97 (0.95-0.99)	0.56 (0.51-0.61)	0.98 (0.96-1.00)	0.97 (0.95-0.99)	0.97 (0.91-1.00)	0.56 (0.51-0.61)	0.67 (0.62-0.72)
**Sex**								

© 2024 Adejumo P et al. *JAMA Network Open.*

**Male**	**Micro-average**	0.98 (0.96-1.00)	0.98 (0.96-1.00)	0.98 (0.96-1.00)	0.96 (0.96-1.00)	0.99 (0.98-1.00)	0.88 (0.84-0.93)	0.69 (0.63-0.76)
	**Macro-average**	0.98 (0.96-1.00)	0.58 (0.53-0.63)	1.00 (1.00-1.00)	0.98 (0.96-1.00)	0.99 (0.93-1.00)	0.58 (0.53-0.63)	0.69 (0.65-0.74)
**Female**	**Micro-average**	0.97 (0.95-0.99)	0.97 (0.95-0.99)	0.97 (0.95-0.99)	0.97 (0.95-0.99)	0.98 (0.96-1.00)	0.86 (0.81-0.91)	0.68 (0.62-0.74)
	**Macro-average**	0.97 (0.95-0.99)	0.57 (0.52-0.62)	0.99 (0.98-1.00)	0.97 (0.95-0.99)	0.98 (0.92-1.00)	0.57 (0.52-0.62)	0.68 (0.64-0.73)
**Ejection Fraction**								
**<40%**	**Micro-average**	0.98 (0.96-1.00)	0.98 (0.96-1.00)	0.98 (0.96-1.00)	0.96 (0.96-1.00)	0.99 (0.98-1.00)	0.88 (0.84-0.93)	0.69 (0.63-0.76)
	**Macro-average**	0.98 (0.96-1.00)	0.58 (0.53-0.63)	1.00 (1.00-1.00)	0.98 (0.96-1.00)	0.99 (0.93-1.00)	0.58 (0.53-0.63)	0.69 (0.65-0.74)
**≥40%**	**Micro-average**	0.97 (0.95-0.99)	0.97 (0.95-0.99)	0.97 (0.95-0.99)	0.97 (0.95-0.99)	0.98 (0.96-1.00)	0.86 (0.81-0.91)	0.68 (0.62-0.74)
	**Macro-average**	0.97 (0.95-0.99)	0.57 (0.52-0.62)	0.99 (0.98-1.00)	0.97 (0.95-0.99)	0.98 (0.92-1.00)	0.57 (0.52-0.62)	0.68 (0.64-0.73)

© 2024 Adejumo P et al. *JAMA Network Open.*

eTable 11. Performance Metrics of Symptom Association NLP Model Across Subgroups. Abbreviations: AUROC, Area under the receiver operating characteristic curve; AUPRC, Area under the precision-recall curves; CI, Confidence Interval; NYHA, New York Heart Association.

Subgroup	Average	Accuracy (95% CI)	Precision (95% CI)	Recall (95% CI)	Specificity (95% CI)	AUROC (95% CI)	AUPRC (95% CI)	F1-Score (95% CI)
**Race**								
**White**	**Micro-average**	0.95 (0.91-0.99)	0.89 (0.83-0.95)	0.91 (0.85-0.97)	0.96 (0.93-1.00)	0.94 (0.89-0.98)	0.83 (0.76-0.90)	0.90 (0.84-0.96)
	**Macro-average**	0.95 (0.91-0.99)	0.87 (0.81-0.94)	0.71 (0.62-0.80)	0.95 (0.90-0.99)	0.83 (0.75-0.90)	0.65 (0.55-0.74)	0.75 (0.67-0.84)
**Black**	**Micro-average**	0.94 (0.90-0.98)	0.88 (0.82-0.94)	0.90 (0.84-0.96)	0.95 (0.92-0.99)	0.93 (0.88-0.97)	0.82 (0.75-0.89)	0.89 (0.83-0.95)
	**Macro-average**	0.94 (0.90-0.98)	0.86 (0.80-0.93)	0.70 (0.61-0.79)	0.94 (0.89-0.98)	0.82 (0.74-0.89)	0.64 (0.54-0.73)	0.74 (0.66-0.83)
**Asian**	**Micro-average**	0.95 (0.91-0.99)	0.89 (0.83-0.95)	0.91 (0.85-0.97)	0.96 (0.93-1.00)	0.94 (0.89-0.98)	0.83 (0.76-0.90)	0.90 (0.84-0.96)
	**Macro-average**	0.95 (0.91-0.99)	0.87 (0.81-0.94)	0.71 (0.62-0.80)	0.95 (0.90-0.99)	0.83 (0.75-0.90)	0.65 (0.55-0.74)	0.75 (0.67-0.84)
**Other**	**Micro-average**	0.94 (0.90-0.98)	0.88 (0.82-0.94)	0.90 (0.84-0.96)	0.95 (0.92-0.99)	0.93 (0.88-0.97)	0.82 (0.75-0.89)	0.89 (0.83-0.95)
	**Macro-average**	0.94 (0.90-0.98)	0.86 (0.80-0.93)	0.70 (0.61-0.79)	0.94 (0.89-0.98)	0.82 (0.74-0.89)	0.64 (0.54-0.73)	0.74 (0.66-0.83)
**Sex**								

© 2024 Adejumo P et al. *JAMA Network Open.*

**Male**	**Micro-average**	0.95 (0.91-0.99)	0.89 (0.83-0.95)	0.91 (0.85-0.97)	0.96 (0.93-1.00)	0.94 (0.89-0.98)	0.83 (0.76-0.90)	0.90 (0.84-0.96)
	**Macro-average**	0.95 (0.91-0.99)	0.87 (0.81-0.94)	0.71 (0.62-0.80)	0.95 (0.90-0.99)	0.83 (0.75-0.90)	0.65 (0.55-0.74)	0.75 (0.67-0.84)
**Female**	**Micro-average**	0.94 (0.90-0.98)	0.88 (0.82-0.94)	0.90 (0.84-0.96)	0.95 (0.92-0.99)	0.93 (0.88-0.97)	0.82 (0.75-0.89)	0.89 (0.83-0.95)
	**Macro-average**	0.94 (0.90-0.98)	0.86 (0.80-0.93)	0.70 (0.61-0.79)	0.94 (0.89-0.98)	0.82 (0.74-0.89)	0.64 (0.54-0.73)	0.74 (0.66-0.83)
**Ejection Fraction**								
**<40%**	**Micro-average**	0.95 (0.91-0.99)	0.89 (0.83-0.95)	0.91 (0.85-0.97)	0.96 (0.93-1.00)	0.94 (0.89-0.98)	0.83 (0.76-0.90)	0.90 (0.84-0.96)
	**Macro-average**	0.95 (0.91-0.99)	0.87 (0.81-0.94)	0.71 (0.62-0.80)	0.95 (0.90-0.99)	0.83 (0.75-0.90)	0.65 (0.55-0.74)	0.75 (0.67-0.84)
**≥40%**	**Micro-average**	0.94 (0.90-0.98)	0.88 (0.82-0.94)	0.90 (0.84-0.96)	0.95 (0.92-0.99)	0.93 (0.88-0.97)	0.82 (0.75-0.89)	0.89 (0.83-0.95)
	**Macro-average**	0.94 (0.90-0.98)	0.86 (0.80-0.93)	0.70 (0.61-0.79)	0.94 (0.89-0.98)	0.82 (0.74-0.89)	0.64 (0.54-0.73)	0.74 (0.66-0.83)

© 2024 Adejumo P et al. *JAMA Network Open.*

eTable 12. Post-Deployment Analysis of NYHA Classification by Ejection Fraction Category. Abbreviations: NYHA, New York Heart Association.

**Ejection Fraction Category**	**Explicit NYHA Class Mention**	**Recategorized NYHA Class**	**Combined NYHA Class after Recategorization**
**Reduced (<40%)**<br>n = 7,287			
NYHA Class I	504 (6.8%)	445 (6.0%)	949 (13.7%)
NYHA Class II/III	814 (11.4%)	573 (7.8%)	1,387 (19.2%)
NYHA Class IV	139 (2.0%)	77 (1.1%)	216 (3.4%)
**Total**	**1,457 (20.2%)**	**1,095 (14.7%)**	**2,552 (35.1%)**
**Preserved (≥40%)**<br>n = 24,769			
NYHA Class I	1,253 (5.2%)	983 (4.0%)	2,236 (9.4%)
NYHA Class II/III	1,472 (6.0%)	1,004 (4.1%)	2,476 (10.4%)
NYHA Class IV	239 (0.9%)	252 (1.0%)	491 (1.1%)
**Total**	**2,964 (12.0%)**	**2,239 (9.2%)**	**5,203 (21.3%)**

© 2024 Adejumo P et al. *JAMA Network Open.*

eReferences

1. Eyre, H. *et al.* Launching into clinical space with medspaCy: a new clinical text processing toolkit in Python. *AMIA Annu. Symp. Proc.* **2021**, 438–447 (2021).
2. Heidenreich, P. A. *et al.* 2022 AHA/ACC/HFSA guideline for the Management of Heart Failure: A report of the American College of Cardiology/American Heart Association joint committee on clinical practice guidelines. *Circulation* **145**, (2022).
3. Raphael, C. *et al.* Limitations of the New York Heart Association functional classification system and self-reported walking distances in chronic heart failure. *Heart* **93**, 476–482 (2007).
4. Caraballo, C. *et al.* Clinical implications of the New York Heart Association classification. *J. Am. Heart Assoc.* **8**, (2019).
5. O'Connor, C. M. *et al.* Efficacy and safety of exercise training in patients with chronic heart failure. *JAMA* **301**, 1439 (2009).
6. The effect of digoxin on mortality and morbidity in patients with heart failure. *N. Engl. J. Med.* **336**, 525–533 (1997).
7. Pitt, B. *et al.* Spironolactone for heart failure with preserved ejection fraction. *N. Engl. J. Med.* **370**, 1383–1392 (2014).
8. Felker, G. M. *et al.* Rationale and design of the GUIDE-IT study. *JACC Heart Fail.* **2**, 457–465 (2014).

© 2024 Adejumo P et al. *JAMA Network Open.*
