# Supplementary material for: Natural Language Processing of Clinical Documentation to Assess Functional Status in Patients With Heart Failure
Source: JAMA Netw Open. 2024 Nov 7;7(11):e2443925. doi: 10.1001/jamanetworkopen.2024.43925 (PMC11544492; doi:10.1001/jamanetworkopen.2024.43925)
Supplement: Supplement 2. — Data Sharing Statement [file jamanetwopen-e2443925-s002.pdf]

Data Sharing Statement

Adejumo. Natural Language Processing of Clinical Documentation to Assess Functional Status in Patients With Heart Failure. *JAMA Netw Open*. Published November 07, 2024. doi:10.1001/jamanetworkopen.2024.43925

Data
Data available: No

Additional Information
Explanation for why data not available: The data cannot be publicly shared as it represents protected health information and sharing data will be a violation of patient privacy.
